# Help! No IOLs for cataract surgery

**Published:** 2013

**Authors:** Elmien Wolvaardt Ellison

**Affiliations:** Editor: *Community Eye Health Journal,* International Centre for Eye Health, London School of Hygiene and Tropical Medicine, London, UK. **editor@cehjournal.org**

**Figure:**
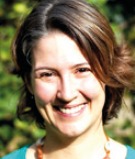
Elmien Wolvaardt Ellison

During a recent visit to Africa, an ophthalmologist told me that his eye department, in a rural part of his country, had recently lost its non-governmental organisation (NGO) support. As a result, he had not performed any cataract operations for several weeks, because the hospital had not ordered the necessary intra-ocular lenses (IOLs). This seemed a very unfortunate state of affairs – but also something others could learn from.

We have therefore invited ophthalmologists and regular *Community Eye Health Journal* contributors in Africa to explain how this situation could be better handled – and avoided – in future. What follows is an edited collection of their responses.

## Boateng Wiafe Ghana

Cataract surgery should be the minimum service offered in any ophthalmic setting, especially when there is a resident ophthalmologist. It takes time to build up a practice and win the confidence of patients. Once this is done one has to make sure that the services are regular.

The ultimate solution is to educate management and encourage them to stock these items in the pharmacy, just like all the other essential items they stock.

In the short term, network with other eye institutions and barter with them. This is what I used to do many years ago when facing similar situations. For example, one institution may have IOLs in abundance, but no sutures or visco-elastic, and may be willing to make an exchange if you have sutures or visco-elastic to spare. Keep management informed so they can officially record the transaction, and ensure that everything you exchange or receive can be used before it reaches the expiry date. You could also ask patients to buy their own IOLs.

## Heiko Phillippin Tanzania

In an ideal world, an NGO will commit to support an eye department for a stated time period. Reducing funding should be discussed in advance and should not happen abruptly. On the other hand, eye departments should try to reduce their need for overseas aid over time by reducing costs and increasing local income, e.g. from consultation fees, health insurance, or local donors.

I would suggest that my colleague develops a business plan for cataract surgery which demonstrates to the hospital management its potential as a feasible procedure. Hospital administrators and finance managers think and talk in a different language from ophthalmologists, and might have a different definition of a feasible procedure. It is worth becoming familiar with the most important objectives and terms of financial management, e.g. by consulting the free financial guide at **www.mango.org.uk**

This business plan could include increasing income by linking with local health insurance schemes or by attracting both affluent and poor patients, with different fee structures for additional services.

Computer registration of patients and services, a separate cash point with invoices and receipts generated from the same database, stock management and standardised procurement procedures should also be considered. With the help of such a business plan, the ophthalmologist might find a new partner which could even be the hospital itself.

## Hannah Faal Nigeria

When we talk about purchasing IOLs, administrators might be thinking some or all of the following.

- What is it? You have never explained or shown it to me, or shown me how it works.
- Your NGOs have been providing them all these years, why have they stopped? Please go back to them.
- I do not know where to buy them.
- The number you need is too small.
- They are not available locally and cannot be bought using a local purchase order (LPO).
- They are too expensive and do not generate revenue for the hospital.
- Patients buy their glasses and dentures; they should buy these too.

Unless ophthalmologists are able successfully to bridge this gap, they and their patients will lose out.

It is worth remembering that an IOL is an item, with a seller and a buyer. I would advise the ophthalmologist to think like an entrepreneur and start a business importing IOLs or similar items. Fix the prices at a level which patients can afford and think about sourcing, importation policy, duty waivers, purchase by LPO, advertising, and so on.

## John Nkurikiye Rwanda

This doctor should use his own initiative. Cataract surgery at a referral hospital is not a free service. It is up to the doctor to convince the management of the hospital to put IOLs on their annual procurement list. For example, in Rwanda, IOLs are on the new list of essential drugs and consumables and there is no reason why they cannot be purchased.

## Susan Lewallen South Africa

Stock keeping and procurement are critical management tasks in an eye care service. Unless they are taken seriously, such ‘crises’ will continue to happen.

**Figure:**
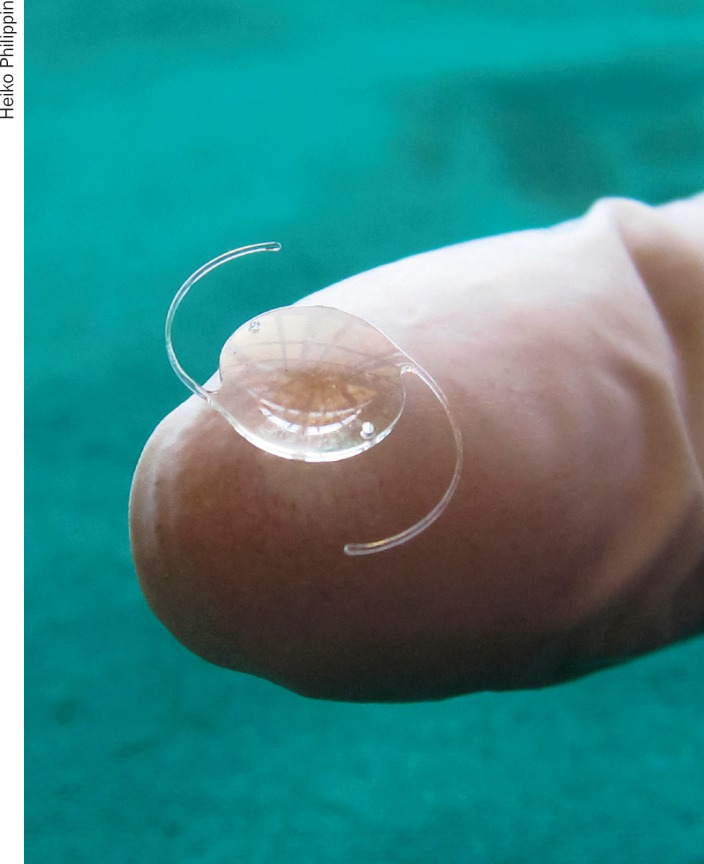
Polymethylmethacrylate (PMMC) intraocular lenses are of high quality and can be procured at affordable prices in low- and middle-income countries.

## References

[B1] Instruments and consumables. Community Eye Health J 2011;24(76):25 PMC3284750 22389557

[B2] Equipment for eye care. Community Eye Health J 2010;23(73):21-22 PMC2975112 21119915

